# Identification and verification of an eight-gene prognostic signature for colorectal cancer based on tumor-associated macrophages

**DOI:** 10.1186/s12885-026-15965-9

**Published:** 2026-04-06

**Authors:** Zhuan Du, Da-Zhong Li, Ze-Long Xu, Qian Li, Yong-You Wu

**Affiliations:** Department of Gastrointestinal Surgery, The Second Affiliated Hospital of Soochow University, No. 1055 Sanxiang Road, Gusu District, Suzhou, 215004 China

**Keywords:** Tumor-associated macrophage, Colorectal cancer, Tumor microenvironment, Prognostic model, Drug response

## Abstract

**Background:**

Tumor-associated macrophages (TAMs), a pivotal component of the tumor microenvironment (TME), play an important role in the development and progression of colorectal cancer (CRC). However, the function of macrophage-related genes (MRGs) in CRC remains poorly understood. This research examined the role of MRGs in CRC.

**Methods:**

MRGs were identified by integrating the gene set that exhibited differential expression between tumor and non-tumor tissues in CRC with the gene set associated with TAMs identified by CIBERSORT. To establish a prognostic signature specifically related to TAMs, a combination of univariate Cox regression and least absolute shrinkage operator analyses was utilized. By incorporating oncoPredict, Tumor Immune Dysfunction and Exclusion, mutation, cancer stem cell index, and metastasis data, we were able to predict various clinical features such as drug sensitivity, chemical sensitivity, and immunotherapeutic response. Furthermore, RNA extraction from CRC tissues enabled the validation of differential expression in prognostic MRGs using quantitative real-time polymerase chain reaction (qRT-PCR).

**Results:**

High TAM infiltration was positively associated with poor prognosis, and the macrophage-related gene (MRG) risk score showed a strong correlation with patient survival. The high-risk group demonstrates better immunotherapy response and six of the eight prognostic genes identified were associated with metastasis, suggesting that the high-risk group may be more susceptible to metastasis. The expression levels of two protective genes, MRPS7 and ORC1, were higher in normal tissues compared to tumor tissues.

**Conclusion:**

Our findings could provide insights into the role of TAMs in predicting CRC prognosis and suggest potential therapeutic targets.

**Supplementary Information:**

The online version contains supplementary material available at 10.1186/s12885-026-15965-9.

## Introduction

Colorectal cancer (CRC) is a major global health issue and the second leading cause of cancer-related deaths worldwide. In 2022, CRC accounted for 16% of cancer diagnoses in both men and women, with a rising incidence rate among young adults [1]. CRC frequently develops insidiously, leading many patients to be diagnosed at advanced stages [2]. Although surgery and chemotherapy are the main methods for CRC treatment, tumor-associated macrophages (TAMs) have become an increasingly important tool and target for the treatment of CRC with the in-depth research of immunotherapy [3]. Prognostic prediction models are commonly used in both clinical practice and research to estimate the likelihood or risk of particular events or outcomes in the future. Hence, investigating the effect of macrophage-associated genes (MRGs) on CRC prognosis and developing a novel prognostic model is essential for the effective management of CRC [4].

In the last decade, TAMs have made significant biological advancements, and their clinical relevance is becoming more apparent [5]. TAMs represent a significant component of the tumor microenvironment (TME) and play important roles in various processes, including angiogenesis, extracellular matrix remodeling, coordination of cancer cell proliferation, metastasis, immune suppression, and resistance to chemotherapy and checkpoint blockade immunotherapy [6]. Macrophage infiltration is commonly linked to adverse prognoses across multiple tumor types. Macrophages can differentiate into M1 or M2 macrophages depending on the prevailing conditions. Higher M1 macrophage levels are associated with a favorable prognosis [7], and increased M2 macrophage levels or a lower lymphocyte-to-monocyte ratio are linked to unfavorable outcomes [8–10]. Consequently, jointly analyzing M1 and M2-related genes is a promising avenue for emerging therapeutic strategies aiming to alter the macrophage phenotype [10, 11].

In this study, we employed the infiltration score of TAMs as a risk factor in CRC. We identified and intersected MRGs with the differentially expressed genes (DEGs) between tumor and normal tissues in CRC, as extracted from the Cancer Genome Atlas Program (TCGA) and the Gene Expression Omnibus (GEO) database. The obtained 125 intersecting genes were subjected to Cox and least absolute shrinkage operator (LASSO) regression analyses, resulting in the identification of eight prognosis-related genes. Subsequently, we validated these genes by GEO cohort and performed pathway enrichment analysis, immune-related functional analysis, mutation, cancer stem cell (CSC) index, Tumor Immune Dysfunction and Exclusion (TIDE), and differential expression analysis in metastatic cancer for the identified prognostic MRGs. Our main objective was to uncover MRGs, develop a prognostic model, and predict drug response using various bioinformatics approaches.

## Materials and methods

### Data source

To collect data on CRC patients, we extracted RNA expression profiles and associated clinical information from the TCGA dataset, focusing on patients diagnosed with colon adenocarcinoma and rectal adenocarcinoma. We removed patients with missing follow-up data and used the remaining 516 patients in our analysis. For validation, we acquired the GSE17538 dataset from the GEO data portal and included 238 patients in our study. Additionally, we used a dataset of CRC with metastasis (GSE41258) to identify the differential expression between primary cancer and metastatic CRC.

### DEGs identification and functional annotation

The “limma” (version 3.54.1) R package was utilized to identify DEGs between tumor and normal tissues in CRC, considering a false discovery rate (FDR) of ≤ 0.05 and a log2 fold change of ≥ 1, which can conduct both differential expression analysis and differential splicing analysis on RNA sequencing data [12].

### Estimation of tumor microenvironment infiltration and identification of MRGs

We employed the CIBERSORT deconvolution method to analyze the gene expression profiles of complex tissues and determine their cell composition. This method utilizes a machine-learning approach called linear support vector regression [13, 14]. The abundance scores of immune cells, including M1 and M2 macrophages, were calculated from patient samples of CRC using the “CIBERSORT” (version 1.03) R package. Specifically, the LM22 signature matrix was used to distinguish macrophage phenotypes. M1 macrophages were characterized by the high expression of markers such as IL-12, iNOS, and CD86, while M2 macrophages were identified by markers including CD163, CD206 (MRC1), and CCL18 [15, 16]. We preserved the results meeting both correlation coefficients > 0.3 with M1 and M2 macrophages and *p*-values < 0.001 and intersected them with DEGs to obtain MRGs.

### Functional and pathway enrichment of MRGs

We used the “clusterProfiler” (version 4.8.1) R package to perform Gene Ontology (GO) and Kyoto Encyclopedia of Genes and Genomes (KEGG) analysis. The “org.Hs.eg.db” (version 3.16.0) R package was utilized as the reference gene set database. We drew plots of the GO and KEGG results using the “ggplot2” (version 3.3.2) R package.

### Construction and validation of a MRG prognostic model

We employed a univariate Cox analysis to confirm the prognostic value of the candidate genes. To construct the risk score model, we used the genes with significant hazard ratios in the multivariate analysis as independent variables. We computed the risk scores for each sample and divided patients into high-risk and low-risk groups according to their risk scores. To assess the prognostic performance of the risk score model, we utilized the “survROC” R package (version 1.0.6) to plot the receiver operating characteristic (ROC) curve. The accuracy of the risk score model in predicting the prognosis of CRC patients was evaluated by calculating the area under the curve of the ROC curve with the same “survROC” R package (version 1.0.6).

To minimize overfitting and improve the reliability of our results, we employed LASSO-Cox regression analysis. We used the “glmnet” R package (version 4.1.6) to perform this analysis [12]. The MRG risk score was calculated by taking the sum of the product of each candidate prognostic gene’s regression coefficient (βi) and its corresponding expression value (Exp). This can be represented as the MRGs risk score = Ʃ (βi * MRGsExp). We stratified the CRC patients into two groups based on the median value of their MRG risk scores, with patients above the median being classified as high-risk and those below as low-risk. The overall survival (OS) curves were plotted using Kaplan–Meier analysis, and ROC analysis over time was performed using the “timeROC” R package (version 0.4). Heatmaps were generated to visualize the correlation between MRG risk scores and candidate genes. Finally, to validate our findings, we conducted further analysis by using the GSE17538 cohort to ensure the robustness and generalizability of our findings.

$$\:{M}{R}{G}{s}\_{r}{i}{s}{k}\:{s}{c}{o}{r}{e}={\sum\:}_{{n}=1}^{{i}}\left({\beta\:}{i}\:{*}\:{M}{R}{G}{s}{E}{x}{p}\right)$$

### Construction of a predictive nomogram

We performed both univariate and multivariate Cox regression (*p* < 0.01) analyses to identify the independent prognostic factors. Subsequently, we created a predictive nomogram utilizing the “rms” R package (version 6.0.1) according to the TAMs signature, clinical stage, and TNM stage. To provide a numerical probability of survival at 1, 3, and 5 years on the basis of independent prognostic factors, we developed a nomogram which serves as a visual tool. We assessed the performance of the nomogram using calibration plots.

### Mutation, CSC index, TIDE, and metastasis

We utilized the mutation annotation format obtained from the TCGA database, processed using the “maftools” R package, to classify somatic mutations in patients with CRC into high- and low-risk groups. We also considered the associations between these risk groups and CSCs. Furthermore, we validated the expression differences of the MRGs in situ carcinoma and metastatic carcinoma using the GSE41258 dataset.

### Drug sensitivity and immunotherapeutic response according to MRGs

To examine the association between gene expression and drug sensitivity, we employed the GDSC2 database and the “oncoPredict” R package (version 0.2). This database provides a link between *in vitro* drug screening and *in vivo* drug and biomarker discovery. The “oncoPredict” R package allows for easy prediction of patient tumor response to a wide range of drugs screened in cancer cell lines, as well as biomarker discovery with and without GLDS [17]. We assessed the sensitivity differences of the low- and high-risk groups to commonly used anticancer drugs by employing the Kruskal–Wallis rank-sum test (*p* < 0.05) [18]. To predict the potential response to immune checkpoint blockade therapy in both groups, we utilized the TIDE algorithm, which is available at http://tide.dfci.harvard.edu/.

### Clinical samples collection, RNA isolation, and quantitative real-time PCR

The Ethics Committee of the Second Affiliated Hospital of Soochow University granted authorization for this study. Tumor tissue samples from patients with CRC and corresponding adjacent normal tissues were collected during surgeries conducted between 2021 and 2023. Ten patients with CRC were enrolled in the study.

(Note: Continue with the existing description of TRIzol, NanoDrop, etc.)

TRIzol Reagent (Cat: 15596026, Invitrogen, CA, USA) was used for the isolation of total RNA. A NanoDrop™ One spectrophotometer (Thermo Scientific, MA, USA) was utilized for the measurement of total RNA and dsDNA concentrations. To generate cDNA templates, the PrimeScript RT Reagent Kit (Cat: RR037A, TAKARA, Kusatsu, Japan) was used for reverse transcription of 500 ng total RNA from each sample. The qPCR primers were synthesized by BioSune Biotech (Shanghai, China), and their sequences are provided in Supplementary Table 1. The Applied Biosystems QuantStudio 7 Flex System (Thermo Scientific, MA, USA) was employed for quantitative real-time PCR using the ChamQ Universal SYBR qPCR Master Mix (Cat: Q711, Vazyme Biotech Co., Ltd., China). Normalization of the relative expression levels of the target genes was performed using the ΔΔCT analysis method with GAPDH as the reference. GAPDH was selected as the internal control due to its verified expression stability across all tumor and normal tissue samples in our cohort. The specificity of the primers for MRPS7, ORC1, and GAPDH was confirmed by observing a single peak in the melting curve analysis for each gene.

### *In vitro* cell experiments

#### Cell culture and transfection

The human CRC cell lines (HCT116 and SW480) and the human monocytic cell line THP-1 were obtained from the American Type Culture Collection (ATCC). HCT116 and SW480 cells were cultured in DMEM, while THP-1 cells were maintained in RPMI-1640 medium. All culture media were supplemented with 10% fetal bovine serum (FBS) and 1% penicillin-streptomycin, and cells were incubated at 37 °C in a humidified atmosphere containing 5% CO2. To investigate the biological functions of MRPS7 and ORC1, overexpression plasmids encoding the full-length sequences of MRPS7 and ORC1 were constructed, while empty vectors were used as negative controls (NC). Transfections were performed using Lipofectamine 3000 (Invitrogen, CA, USA) according to the manufacturer’s protocol. The overexpression efficiency in HCT116 and SW480 cells was subsequently verified using qRT-PCR prior to functional assays.

#### Cell proliferation assay.

The proliferation ability of CRC cells was assessed using the Cell Counting Kit-8 (CCK-8) assay. HCT116 and SW480 cells transfected with overexpression plasmids (OE-MRPS7, OE-ORC1) or empty vectors (Control) were seeded into 96-well plates at a density of 2,000 cells per well. At specified time points (24, 48, 72, and 96 h), 10 µL of CCK-8 reagent was added to each well. After an additional incubation for 2 h at 37 °C in the dark, the optical density (OD) value was measured at 450 nm using a microplate reader.

#### Macrophage induction and Transwell co-culture assay

To investigate the crosstalk between CRC cells and macrophages, a Transwell co-culture system with a 0.4 μm pore size (Corning, NY, USA) was employed. Initially, THP-1 monocytes were induced to differentiate into macrophages by treatment with 100 ng/mL phorbol 12-myristate 13-acetate (PMA) for 24 h. These differentiated macrophages were then seeded into the lower chambers of the Transwell plates. Subsequently, HCT116 cells transfected with OE-MRPS7, OE-ORC1, or Control vectors were seeded into the upper chambers. After 48 h of continuous co-culture, the macrophages in the lower chambers were harvested for downstream phenotypic analysis.

#### Flow cytometry and Western blot analysis

The polarization state of the co-cultured macrophages was evaluated. For flow cytometry, the harvested macrophages were washed, resuspended in PBS, and co-stained with fluorescently conjugated antibodies against M2 macrophage surface markers (F4/80 and CD206) in the dark. The stained cells were then analyzed using a flow cytometer. For Western blot analysis, total proteins were extracted from the macrophages using RIPA lysis buffer, quantified by the BCA assay, and separated by SDS-PAGE. After transferring the proteins to PVDF membranes and blocking with 5% non-fat milk, the membranes were incubated overnight at 4 °C with primary antibodies against CD206 and β-actin. Following incubation with HRP-conjugated secondary antibodies, the protein bands were visualized using an enhanced chemiluminescence (ECL) detection system.

### Statistical analysis

Statistical analyses were conducted using the R software version 4.2.2. To compare the two groups, we used the Wilcoxon signed-rank test. The Kruskal-Wallis test was utilized to compare three or more groups. For survival analysis, we employed the Kaplan–Meier method with the log-rank test. To construct a prognostic model for CRC patients, we utilized LASSO as well as univariate and multivariate Cox regressions. A *p*-value below 0.05 was considered statistically significant.

## Results

### Identification of DEGs and MRGs

**Fig. 1:**
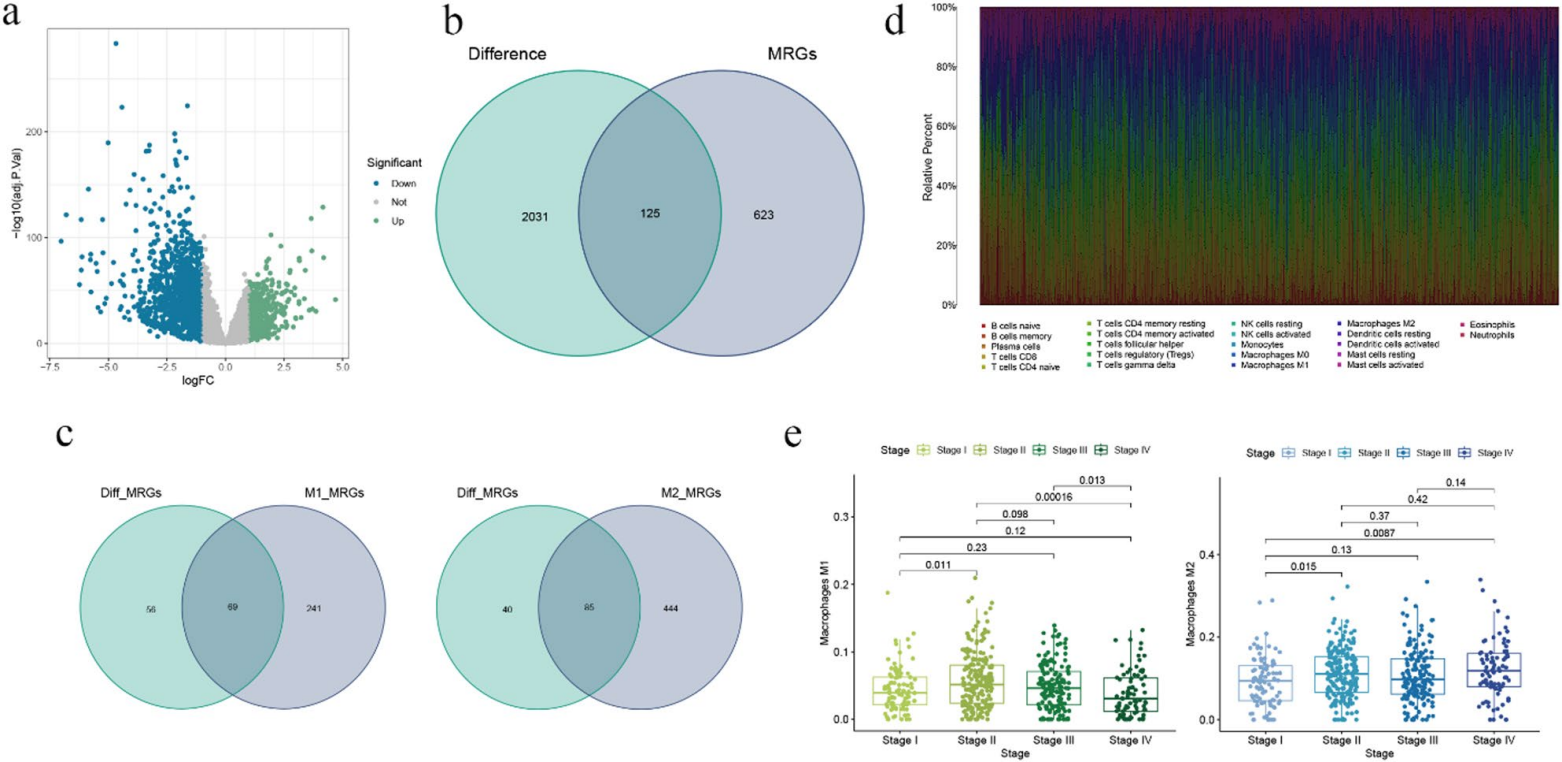
Identification of DRGs and MRGs. (**a**) Expression of differential genes; (**b**) Intersection between differential genes and MRGs; (**c**) Number of M1 and M2 macrophages among MRGs; (**d**) Proportion of immune cell infiltration; (**e**) Differential expression of M1 and M2 macrophages in CRC patients at different stages

Differential expression analysis was performed on CRC patients using the “limma” package (Fig. 1a). From the intersecting gene set obtained (Fig. 1b), we identified 125 genes, out of which 69 were associated with M1 macrophages, 85 were associated with M2 macrophages, and 29 genes were associated with both M1 and M2 macrophages, satisfying the aforementioned criteria (Fig. 1c).

### Infiltration of immune cell in colorectal cancer TME

The TME of CRC is known to harbor a significant number of TAMs, whose critical role in tumor development and metastasis cannot be overstated. Through the application of CIBERSORT methodology, we were able to display the proportions of immune cells within CRC tissue (Fig. 1d), and employed bar graphs (Figs. 1e) to illustrate the differences in M1 and M2 macrophage infiltrations at different stages of tumor progression. As demonstrated by our findings, both M1 and M2 macrophages exhibit significant variation between Stages I, II, and IV, underscoring their close association with the lymph node status and metastatic potential of CRC patients (Table 1).

**Table 1:** Baseline characteristics of CRC patients in the TCGA and GEO cohorts

Characteristic	TCGA Cohort (*n* = 516)	GSE17538 (*n* = 238)	GSE41258 (*n* = 253)
Age, years			
Median (Range)	68 (31–90)	66 (40–85)	62 (22–92)
Gender, n (%)			
Male	285 (55%)	130 (55%)	146 (57.7%)
Female	231 (45%)	108 (45%)	107 (42.3%)
TNM Stage, n (%)			
I	60 (12%)	30 (13%)	45 (17.8%)
II	160 (31%)	70 (29%)	78 (30.8%)
III	185 (36%)	80 (34%)	63 (24.9%)
IV	111 (21%)	58 (24%)	67 (26.5%)
Survival Status, n (%)			
Alive	320 (62%)	160 (67%)	138 (54.5%)
Dead	196 (38%)	78 (33%)	115 (45.5%)

### Construction and verification of the prognostic model

**Fig. 2:**
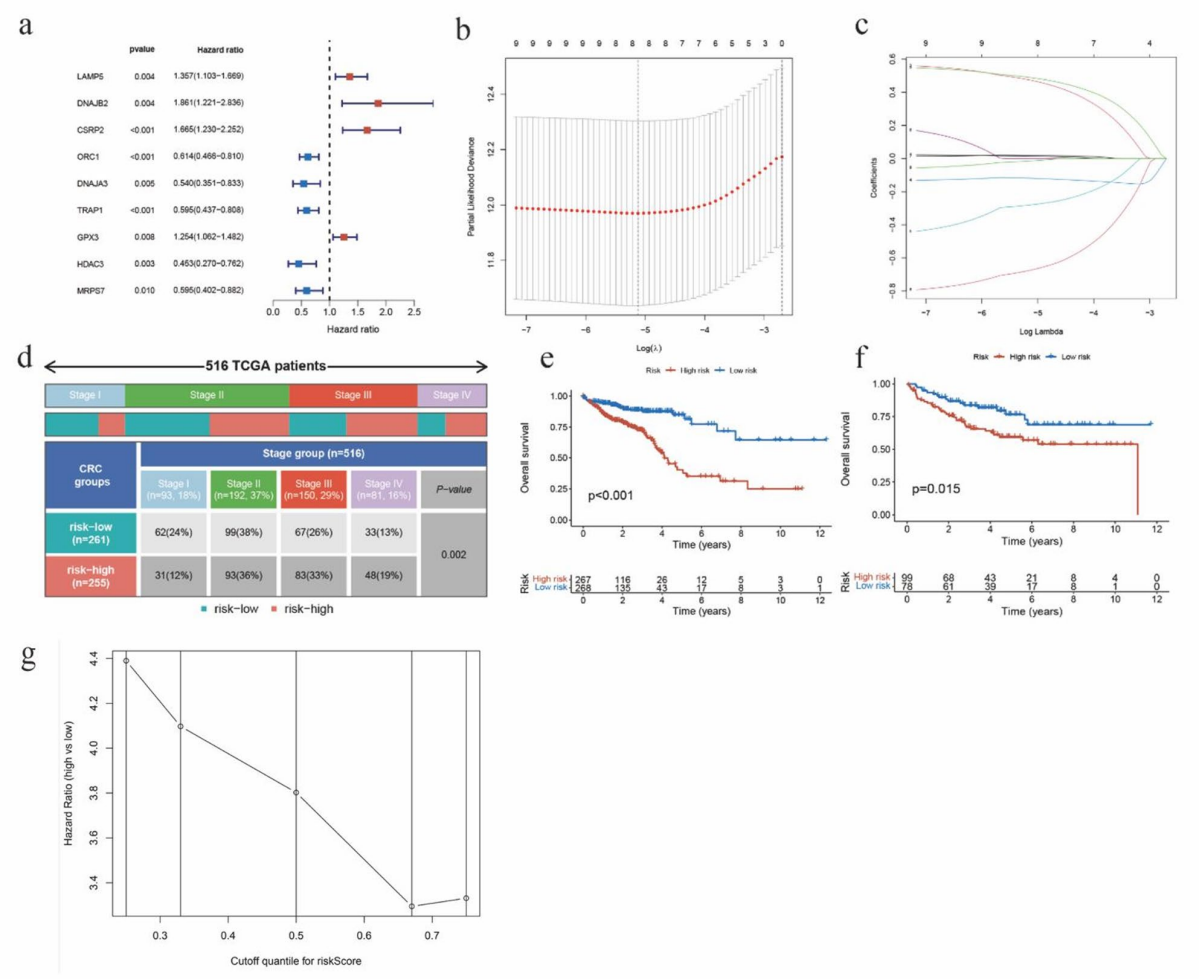
Construction of prognostic model and K-M analysis. (**a**) Univariate Cox analysis of MRGs. (**b**) Cross-validation for tuning parameter selection in the LASSO regression. The partial likelihood deviance is plotted against log(λ). The left vertical dashed line indicates the minimum partial likelihood deviance (λ_min_), and the right vertical dashed line indicates the 1-standard error criterion (λ_1se_). We selected λ_min_ for model construction. (**c**) LASSO coefficient path plot showing the shrinkage of gene coefficients as a function of log(λ). (**d**) Staging and risk status of TCGA cohort used for model construction. (**e**) Overall survival analysis of two patient groups in the TCGA cohort. (**f**) Overall survival validation analysis of two groups in the GEO cohort. (**g**) Sensitivity analysis of the risk score grouping strategy using different cutoffs (25%, 33%, 50%, 67%, and 75% quartiles) to verify model robustness

To assess the prognostic value of the variables, we performed a univariate Cox regression analysis on the MRGs of TCGA CRC patients, and nine genes were found to have p-values less than 0.01 (Fig. 2a). We subsequently used LASSO regression analysis to further narrow down the selection of genes from the nine candidates mentioned above, and identified hub genes and the optimal value of the minimum logarithm (lambda) through ten-fold cross-validation (Figs. 2b, c). The prognostic feature model for the eight prognostic MRGs was constructed according to the expression levels and coefficients of each gene. To calculate the risk score for each patient, we used the following formula: risk score = (0.0144516275927198 * Exp of LAMP5) + (0.473035329316953 * Exp of DNAJB2) + (0.488891793746562 * Exp of CSRP2) + (0.0199908686522824 * Exp of GPX3) - (0.118586826757322 * Exp of ORC1) - (0.279689763205497 * Exp of DNAJA3) - (0.673721052119672 * Exp of HDAC3) - (0.0142709783533031 * Exp of MRPS7). LAMP5, DNAJB2, CSRP2, and GPX3 were identified as risk genes, and ORC1, DNAJA3, HDAC3 and MRPS7 were identified as protective genes.

We then divided the TCGA and GEO cohort into high-risk and low-risk groups according to the median risk score of macrophages, and the GEO cohort was used to verify the results. We selected the median risk score as the cutoff to categorize patients into high- and low-risk groups. This grouping strategy, independent of survival outcomes, avoids potential bias arising from arbitrarily selecting optimal cutoffs and ensures relatively balanced sample sizes between the two groups, thereby enhancing the reproducibility of the model.

### Sensitivity analysis of the risk score grouping strategy

**Fig. 3:**
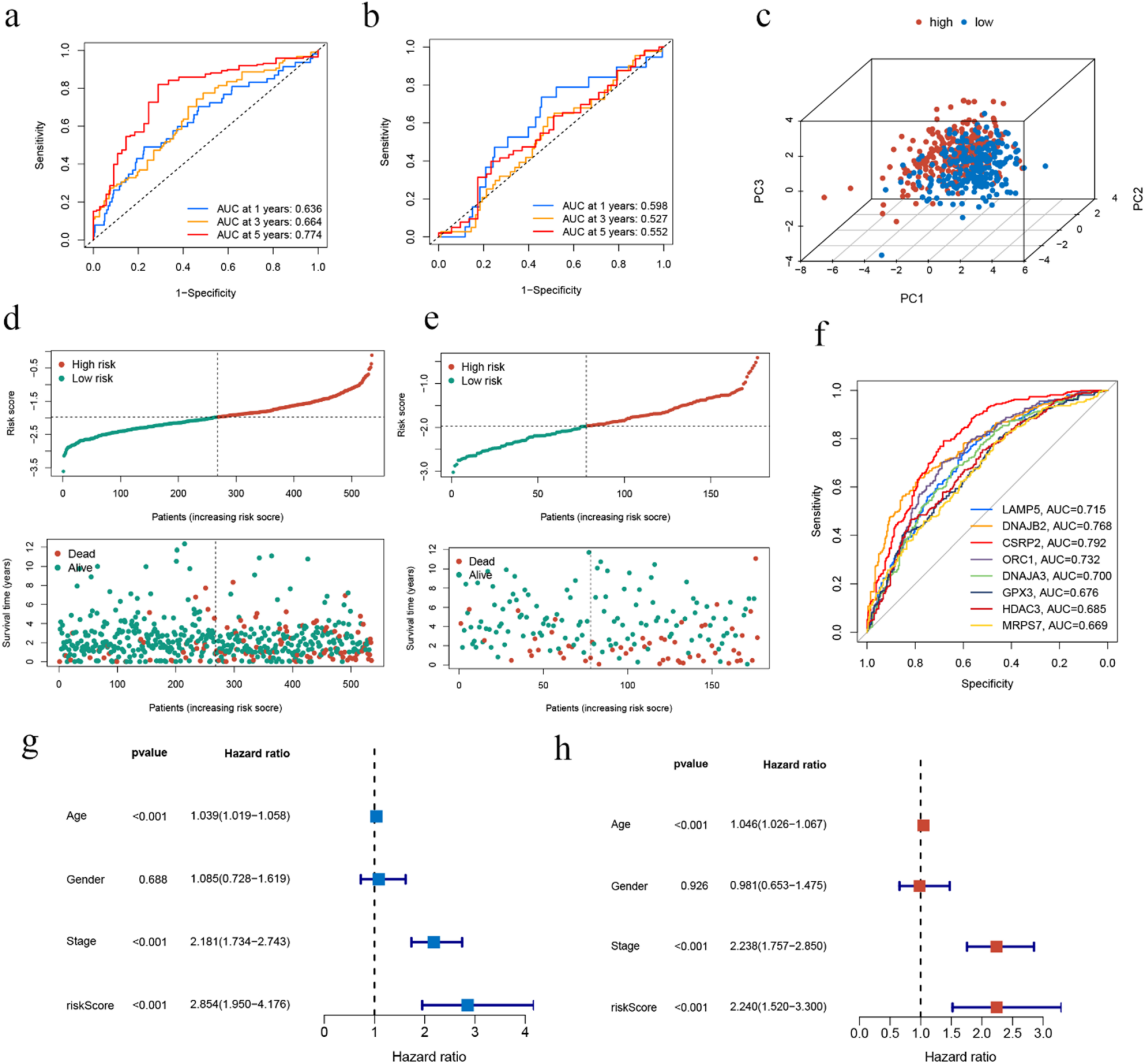
ROC curves and validation of TCGA and GEO cohorts. (**a**) Time-dependent ROC curves in the TCGA group; (**b**) Time-dependent ROC curves in the GEO group; (**c**) PCA analysis of the two patient groups. (**d**) Distribution and survival status of each patient in the TCGA group; (**e**) Distribution and survival status of each patient in the GEO group; (**f**) ROC curves of the 8 genes comprising the model; (**g**) Univariate Cox analysis of risk score and other clinical factors; (**h**) Multivariate Cox analysis of risk score and other clinical factors

**Fig. 4:**
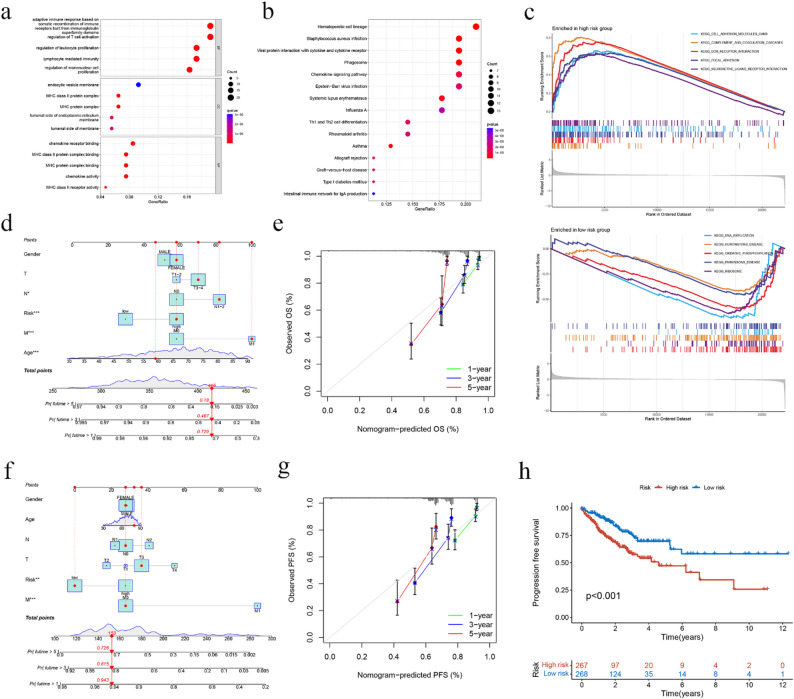
GO/KEGG analysis and nomo plots for OS and PFS. (**a**) GO enrichment analysis of MRGs; (**b**) KEG enrichment analysis of MRGs; (**c**) GSEA enrichment analysis of the 8 prognostic MRGs in two groups; (**d-e**). Nomo plots for overall survival and calibration curves for 1-, 3-, and 5-year OS; (**f-g**) Nomo plots for progression-free survival and calibration curves for 1-, 3-, and 5-year PFS; h. Kaplan-Meier survival curves comparing the progression-free survival (PFS) between the high-risk and low-risk groups

To verify the robustness of our risk score grouping strategy, we further conducted a sensitivity analysis using different risk score cutoffs (25th, 33rd, 50th, 67th, and 75th percentiles). The results demonstrated that across these different cutoffs, the hazard ratios between the high-risk and low-risk groups were consistently greater than 1, with consistent effect directions. These findings indicate that the prognostic model exhibits good stability regarding cutoff selection and is not dependent on a single threshold (Fig. 2g).We conducted survival analysis of the two patient groups and found that the overall survival rate of the high-risk group was significantly lower than that of the low-risk group, as indicated by the Kaplan-Meier (K-M) survival curve (Figs. 2e, f). Furthermore, the area under the ROC curve at 1, 3, and 5 years were 0.636, 0.664, and 0.774, respectively, indicating a moderate predictive accuracy of the model (Fig. 3a). These finding was showed in the GEO cohort as well (Figs. 3b). We then performed principal component analysis (PCA) on the RNA information of the two patient groups. The resulting three-dimensional plot (Fig. 3c) demonstrated that the high-risk and low-risk groups could be differentiated according to the expression levels of these eight genes. We also included a risk curve and a survival status graph (Figs. 3d, e). Furthermore, we conducted multivariate ROC analysis on the eight prognosis-related MRGs to evaluate their predictive capabilities. Among these genes, CSRP2 exhibited the highest predictive power (Fig. 3f). We likewise treated the risk score derived from the eight MRGs as an independent variable and combined it with other clinical features for both univariate and multivariate Cox regression analyses. The risk ratio and 95% confidence interval were 2.853 (1.950–4.176) and 2.240 (1.520–3.30), respectively (Figs. 4g, h).

After analyzing the data from the cohorts, we confirmed that a risk score, which considers eight specific genes and tumor stages, can serve as an independent prognostic factor for patients. These findings suggest that the risk score could serve as a reliable predictor of patient outcomes.

### Functional downstream analyses of MRGs using GO and KEGG

To gain insights into the biological functions and pathways of the genes identified above, we performed a comprehensive analysis of MRGs and found a significant enrichment in the adaptive immune response based on the somatic recombination of immune receptors built from immunoglobulin superfamily domains and regulation of the T cell activation pathway in the GO database (Fig. 4a). Moreover, the MRGs were significantly enriched in hematopoietic cell lineage and staphylococcus aureus infection in the KEGG database (Fig. 4b). We subsequently conducted a gene set enrichment analysis (GSEA) to assess the enrichment of prognostic MRGs. The results showed the top five significant enrichment pathways in the high and low-risk groups (Fig. 4c). Specifically, the high-risk group exhibited a substantial enrichment in the complement and coagulation cascades and ECM receptor interaction, and the low-risk group demonstrated the most noteworthy enrichment in the DNA replication pathways.

### Establishment of a nomogram for CRC patients

We created a nomogram to serve as a reference model for predicting the 1-, 3-, and 5-year survival rates of patients with CRC. This nomogram is an intuitive and practical tool that can assist clinicians in making informed treatment decisions for their patients. We constructed a calibration curve to assess the level of concordance between the predicted survival rates obtained from the nomogram and the observed survival rates in the patient cohort (Figs. 4d, e). This analysis serves as a crucial step in verifying the accuracy and reliability of the nomogram in predicting patient outcomes. We also established another nomogram and the corresponding calibration curves to predict PFS (Figs. 4f, g). Finally, we compared progression-free survival (PFS) for the high-risk/low-risk groups, with a p-value < 0.001(Fig. 4h).

### Association between MRGs and TME, mutation, TIDE, CSC index, and metastasis

**Fig. 5:**
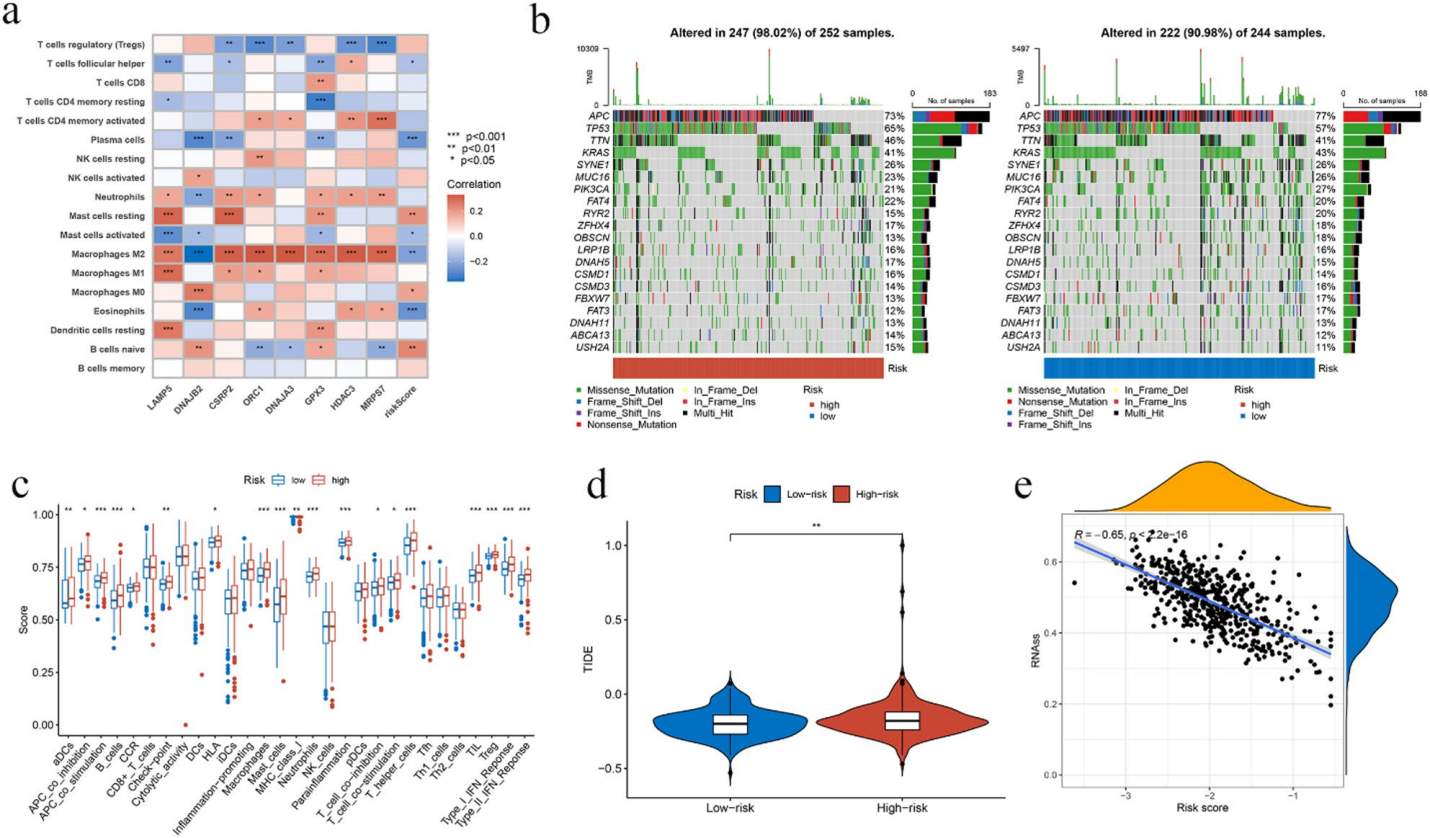
Multifaceted analysis between high- and low-risk groups. (**a**) Relationship between prognostic MRGs and immune cells; (**b**) Comparison of gene mutations between the two patient groups; (**c**) Differences in immune function; (**d**) Comparison of TIDE scores; (**e**) Comparison of cancer stem cell (CSC) characteristics. (*p* < 0.05 *; *p* < 0.01 **; *p* < 0.001 ***)

First, we investigated the relationship between immune cell enrichment and the eight genes in the model. Figure 5a demonstrates a significant correlation between the eight genes and immune cell enrichment. All eight genes showed strong associations with M1 and M2 macrophages. Specifically, DNAJB2 exhibited a negative correlation with M2 macrophages, LAMP5 showed positive correlations with both M1 and M2 macrophages, and CSRP2, which exhibited the strongest predictive ability, showed a strong association with M2 macrophages.

To examine the distributional changes in somatic mutations in CRC patients, we first searched the TCGA database. Both groups of genes exhibited relatively high mutation rates, but we observed that the mutation rates of TP53 and TTN were lower in the low-risk group compared to those in the high-risk group (Figs. 5b, c).

We then compared the differences in immune-related functions and TIDE between the high-risk and low-risk groups and investigated the relationship between the CSC index and risk scores. As shown in Fig. 5c, significant differences occurred in the immune functions of the two groups. Additionally, the high-risk group exhibited a better response to immunotherapy compared to the low-risk group. The CSC index also significantly decreased with increasing risk scores (Figs. 5d-e).

**Fig. 6:**
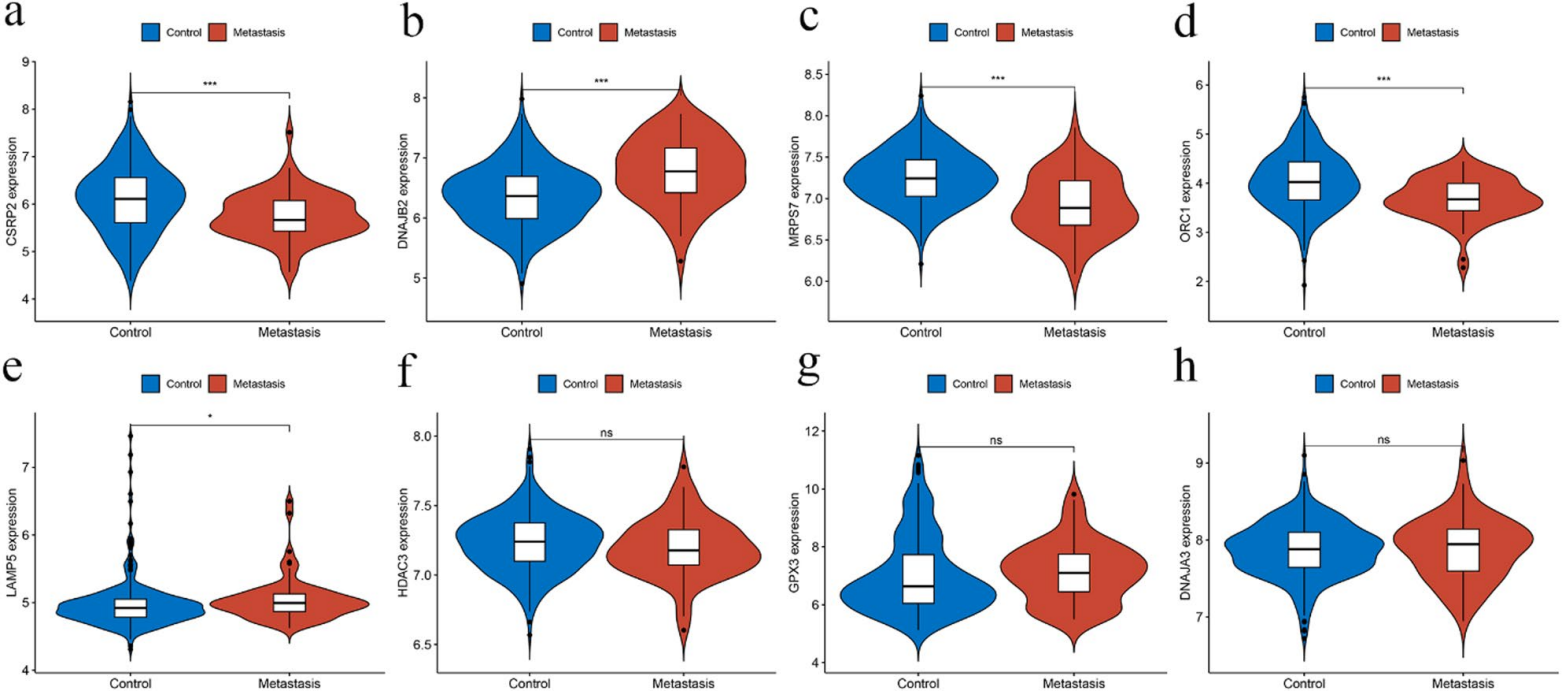
Expression differences of the eight prognostic MRGs between primary and metastatic CRC tissues. (**a–h**) Violin plots displaying the expression levels of CSRP2, DNAJB2, MRPS7, ORC1, LAMP5, HDAC3, GPX3, and DNAJA3 in the control (primary CRC) group (*n* = 186) compared to the metastatic group (*n* = 67). The width of the violin represents the probability density of the data, and the internal box plots indicate the median and interquartile range. (*p* < 0.05 *; *p* < 0.001 ***)

Finally, we selected the GSE41258 dataset to validate the expression of the eight prognostic-related MRGs in metastasis CRC patients. The patients in the dataset were divided into the metastasis and control groups, in which the control group did not experience cancer metastasis. The results revealed that among the eight MRGs, CSRP2, DNAJB2, MRPS7, ORC1, and LAMP5 exhibited differential expression patterns in metastatic tissues. These findings suggest they are potential candidates for metastasis-related investigation, warranting further validation to establish their roles as drivers of metastasis. (Fig. 6).

### Prediction of drug sensitivity

**Fig. 7:**
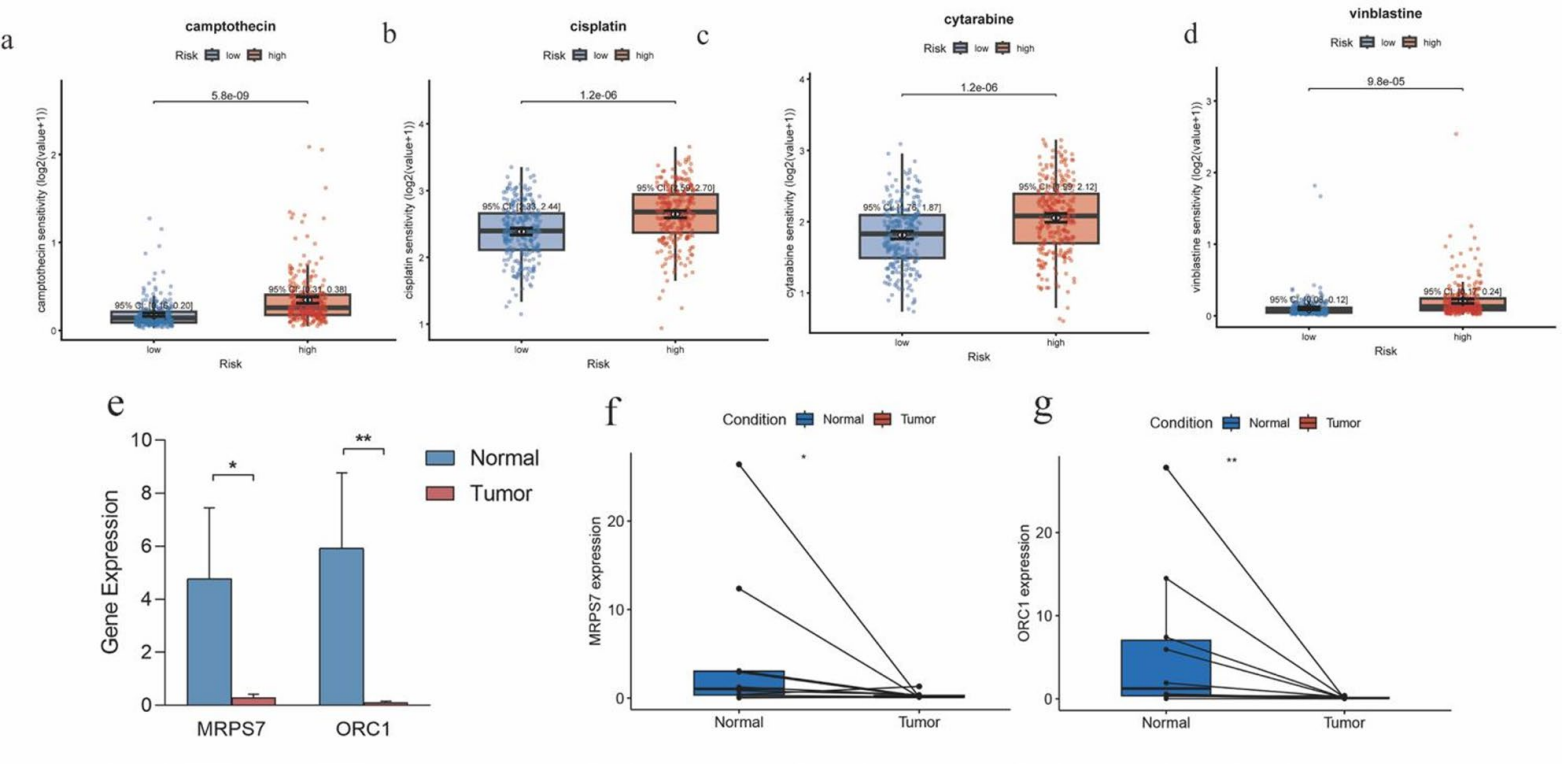
Drug sensitivity analysis and qRT-PCR validation. (**a-d**) Differential analysis of camptothecin, cisplatin, cytarabine, and vinblastine sensitivities between the low-risk and high-risk groups. Data are presented as box plots with median and interquartile range. (**e**) Bar graph showing qRT-PCR quantification analysis of MRPS7 and ORC1 expression in 10 paired clinical samples. Error bars represent the standard deviation (SD). (**f-g**) Paired differential analysis of MRPS7 and ORC1. (*p* < 0.05; *p < 0.01; **p < 0.001)

We examined the variations in drug sensitivity between the low-risk and high-risk groups. This investigation shed light on the potential differences in the treatment responses of these two groups and highlights the potential implications of our findings for personalized therapeutic approaches. We utilized the “oncoPredict” package to evaluate the drug sensitivity of frequently prescribed chemotherapy medications in patients with CRC (Fig. 7a-d). Our analysis revealed that the treatment outcomes of camptothecin, cisplatin, cytarabine and vinblastine varied significantly between the low-risk and high-risk groups. These findings suggest that our risk stratification model may be valuable in guiding personalized treatment decisions for patients with CRC. These results further highlight that the high-risk group may exhibit heightened sensitivity to drug treatment.

### Validation of MRPS7 and ORC1 expression and function

In our developed risk models, MRPS7 and ORC1 were identified as key prognostic genes demonstrating a protective effect. To experimentally verify these bioinformatics findings, we performed a preliminary validation using qRT-PCR on a local cohort of 10 paired CRC and adjacent normal tissue samples (Fig. 7e-g). Despite the limited sample size, the results were consistent with the transcriptomic data from TCGA that both MRPS7 and ORC1 exhibited significantly higher expression levels in normal tissues compared to tumor tissues (*p* < 0.05).

**Fig. 8:**
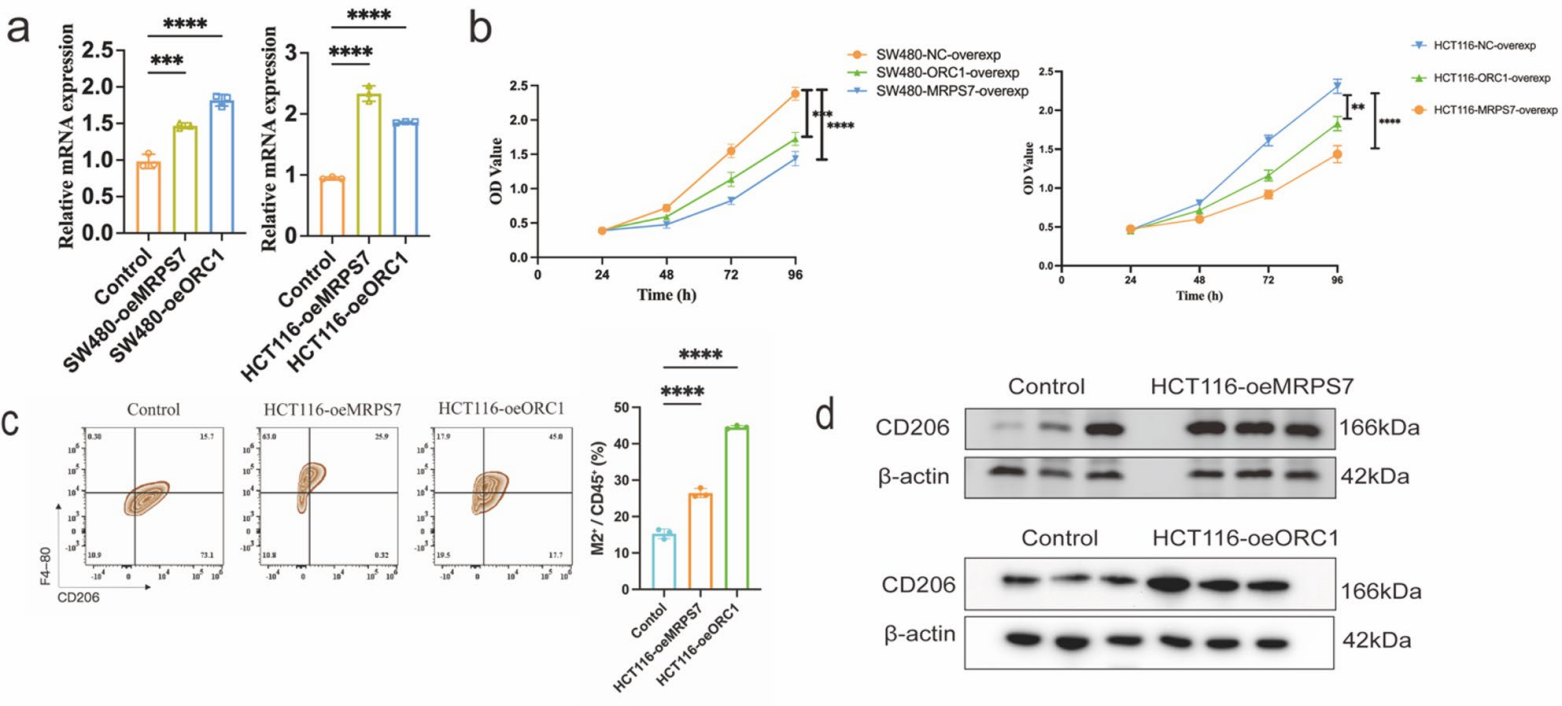
*In vitro* validation of MRPS7 and ORC1 function. (**a**) Verification of MRPS7 and ORC1 overexpression efficiency in SW480 and HCT116 cells by qRT-PCR. (**b**) The effect of MRPS7 and ORC1 overexpression on the proliferation of SW480 and HCT116 cells was assessed by CCK-8 assay at 24, 48, 72, and 96 h. (**c**) Flow cytometry analysis of M2 macrophage polarization (CD206⁺F4/80⁺) in a Transwell co-culture system with HCT116 cells. (**d**) Western blot analysis of the M2 macrophage marker CD206 protein expression levels in macrophages after co-culture with HCT116 cells. Data are presented as mean ± SD. (*p < 0.05, **p < 0.01, ***p < 0.001, ****p < 0.0001)

To further explore the biological functions of these genes, we conducted *in vitro* functional assays. First, we utilized overexpression plasmids to upregulate MRPS7 and ORC1 expression in two CRC cell lines, SW480 and HCT116. Following the confirmation of successful upregulation via qRT-PCR (Fig. 8a), the CCK-8 assay results demonstrated that the overexpression of either gene significantly inhibited cell proliferation in both cell lines (Fig. 8b). Furthermore, we investigated the crosstalk between CRC cells and the immune microenvironment using a Transwell co-culture system. Both flow cytometry and Western blot analyses consistently revealed that co-culturing with HCT116 cells overexpressing MRPS7 or ORC1 significantly induced the polarization of macrophages towards the M2 phenotype, as evidenced by the increased expression of the M2 marker CD206 (Fig. 8c, d). These combined results—clinical expression trends and *in vitro* functional data—provide multidimensional insights into the biological roles of MRPS7 and ORC1 in CRC progression.

## Discussion

Over the past few years, investigations into TME have uncovered numerous factors that contribute to tumor progression and metastasis [10, 19]. TAMs, as an important component of TME, play a crucial role in CRC. TAMs are ubiquitous in numerous types of tumors and have been demonstrated to play a critical role in promoting tumor growth, invasion, metastasis, and drug resistance. Owing to their ubiquity and functional importance within the TME, TAMs represent a promising therapeutic target for innovative cancer treatments [20, 21]. Generally, M1 macrophages can respond to danger signals triggered by bacterial products or IFN-γ, recruiting and activating cells of the adaptive immune system. They are characterized by the expression of inducible nitric oxide synthase, reactive oxygen species, and IL-12 [20, 22]. equipping them to promote inflammation and mount effective immune responses. By contrast, M2 macrophages are characterized by high levels of scavenger receptors, IL-10, IL-1β, VEGF, and matrix metalloproteinases [5, 10, 20], promoting tissue repair, angiogenesis, and immunomodulation. However, excessive M2 polarization can lead to immunosuppression and the promotion of tumor growth and metastasis. In our investigation, we developed a prognostic model utilizing the LASSO-Cox method based on eight TAM-related genes.

Numerous researches have reported a complex interplay between TAMs and CRC cells. Metastatic CRC is enriched with MRC1 + CCL18+ M2 macrophages [23], and a higher M1:M2 density ratio within the TME is associated with improved cancer-specific survival [24]. Previous work by Zhao et al. demonstrated that tumor-derived exosomal miR-934 can drive CRC liver metastasis by modulating communication between CRC cells and TAMs [25], while Zheng et al. found that LINC00543 promotes metastasis by inducing M2 polarization [26]. recent studies have highlighted the critical role of extracellular vesicles in this crosstalk. For instance, Zhang et al. demonstrated that M2 exosome-derived lncRNAs drive metabolic reprogramming in CRC cells, a mechanism highly relevant to the TAM-metastasis axis observed in our study [27]. Similarly, Lv et al. reinforced the plausibility of vesicle-mediated mechanisms, suggesting that broader vesicle biology could underlie the associations we found between MRGs and tumor progression [28]. From a translational perspective, Yang et al. recently demonstrated TME-mediated exosomal immunomodulation, suggesting that our macrophage-centered signature might not only serve as a prognostic tool but could also inform future exosome-based TME engineering and delivery approaches [29].

LAMP5 is one of the top 10 genes associated with poor CRC prognosis [30]. It belongs to the lysosome-associated membrane protein family and has been implicated in the formation of metastasis in gastric cancer. Our findings consistently demonstrate a significant association between LAMP5 expression and metastasis CRC [31]. Therefore, the function of LAMP5 in CRC metastasis needs to be validated further. Activated M2 macrophages, which represent a dominant group in the TME, are negatively correlated with the overall survival of CRC patients [10, 32]. In our study, we identified seven out of the eight genes associated with M2 TAMs. (1) CSRP2 notably plays a crucial role in inhibiting CRC aggressiveness and metastasis through the Hippo, ERK, and PAK signaling pathways [33]. (2) DNAJB2 (HSJ1), functions as a co-chaperone regulatory factor of Hsp70 and is predominantly expressed in the nervous system [34]. (3) MRPS7 serves as a crucial 12 S ribosomal RNA-binding subunit of the small mitochondrial ribosome, playing a vital role in the assembly of the small ribosomal subunit. Defects in MRPS7 have been linked to abnormalities in the respiratory chain complexes I, III, and IV, which have been observed in both fibroblasts and the liver. These mutations in MRPS7 result in the destabilization of the protein’s tertiary structure, leading to impaired RNA binding capacity. Consequently, this disruption not only causes a decrease in the protein’s levels but also impacts the integrity of the entire mitochondrial ribosomal small subunit. As a result, these molecular alterations can contribute to the development of various diseases [35–37]. (4) ORC1 plays a crucial role in various fundamental processes within eukaryotes, including replication, heterochromatin formation, telomere maintenance, and genomic stability. Additionally, during meiosis, orc1-nucleosome contacts serve to safeguard the boundaries of rDNA [38]. Recently, Wang et al. identified ORC1 as a key gene in a mitochondrial metabolism-related signature for CRC [39]. Our study complements this finding by further linking ORC1 expression to the immune microenvironment and TAM infiltration.5) HDAC3, a known malignant marker for CRC, is negatively correlated with miR-296-3p and positively correlated with TGIF1. Through the microRNA-296-3p/TGIF1/TGFβ axis, HDAC3 promotes the progression of CRC [40]. 6) GPX3 plays a critical role in platinum resistance in CRC cells. Methylation of GPX3 leads to decreased expression, resulting in increased sensitivity to oxaliplatin and cisplatin [41]. Recent studies by Haumaier et al. and Mekala et al. have demonstrated methylation-driven transcriptional changes in colitis-associated CRC [42, 43]. Integrating such epigenetic profiling could refine our macrophage-related model by uncovering how methylation patterns modulate these key MRGs. Furthermore, Yu et al. positioned TAM-related genes within a broader network of CRC drivers, highlighting how tumor-intrinsic oncogenic pathways intersect with the microenvironmental regulators identified in our eight-gene signature [44]. 7) Finally, DNAJA3 belongs to the heat shock protein 40 family, and is involved in maintaining mitochondrial DNA integrity and mitochondrial membrane potential. DNAJA3 regulates the migration and invasion of gastric cancer cells. Loss of DNAJA3 may serve as an adverse prognostic factor in gastric cancer [45].

Analysis of TIDE scores and drug sensitivity revealed a complex landscape. The high-risk group exhibited greater immune dysfunction and exclusion yet paradoxically showed enhanced sensitivity to chemotherapeutic agents like cisplatin and camptothecin. While chemotherapy can sometimes reverse TAM polarization [46–48], we must also consider resistance mechanisms. Extracellular vesicle-mediated resistance is a growing concern; Nittayaboon et al. and Ramalingam et al. have elucidated how vesicle cargoes can confer resistance to agents like camptothecin and cisplatin [49, 50]. Therefore, the actual clinical response may depend on the balance between TAM-associated sensitivity and potential vesicle-mediated resistance mechanisms.

Regarding immunotherapy, our analysis links high-risk scores with poorer survival yet potentially better predicted immunotherapy responses (higher TIDE scores). This apparent paradox likely reflects a hot but exhausted tumor microenvironment, where high immune infiltration is counterbalanced by significant dysfunction. Wang et al. and Huang et al. have characterized similar microenvironment-immunotherapy interactions, suggesting our findings are consistent with local TME phenomena distinct from systemic pathologies [51, 52]. Additionally, anchoring our findings within existing interventions, as discussed by Tong et al. helps position our signature alongside established immunometabolic targets [53]. We also observed differential mutation patterns (e.g., TP53, TTN) between risk groups, which may be linked to chronic inflammation reshaping tumor suppressor landscapes, as discussed by Ning et al. [54]. By stratifying patients according to these MRGs, we can better identify those who might benefit from immunotherapy [55].

## Limitations

Despite these promising findings, our study has several limitations that should be acknowledged. First, the sample size of our local clinical validation cohort (*n* = 10) is relatively small due to the retrospective nature of the collection. While our qRT-PCR results were consistent with large-scale public datasets (TCGA and GEO) and supported by our *in vitro* cell experiments, they should be interpreted as a preliminary validation. Large-scale, multi-center prospective studies are needed to robustly confirm these markers. Second, our model focuses on somatic transcriptomics and does not account for germline susceptibility factors, which Maedeh Barahman et al. noted are crucial for shaping CRC risk [56]. Third, although we presented a nomogram, we did not perform decision-curve analysis or calculate reclassification indices in this study. Finally, we acknowledge that the preprocessing of retrospective data from TCGA and GEO might introduce analytic biases [57]. Future studies incorporating *in vivo* animal models are required to fully elucidate the mechanism. In conclusion, our study identified eight MRGs that are crucial in stratifying CRC patients and predicting their response to immunotherapy. Our analysis of TIDE and immune function comparison confirms the necessity of this stratification, which can improve the success rate of immunotherapy and patient survival. These genes can serve as potential predictive indicators for treatment outcomes, guiding subsequent chemotherapy and follow-up care. Additionally, this work provides valuable insights into the potential role of TAMs in tumor development and progression, and offers the possibility of developing targeted therapies for CRC patients on the basis of modulating TAM activity. Overall, our findings have important clinical implications for improving patient outcomes and advancing CRC knowledge.

## Conclusion

In this study, we identified a set of MRGs that are associated with TAMs in CRC. Our findings suggest that these genes could be used to develop a prognostic model for predicting patient outcomes and may assist in risk stratification and hypothesis generation for treatment selection.

## Supplementary Information

Supplementary Material 1.

Supplementary Material 2.

## Data Availability

Publicly available datasets were analyzed in this study. This data can be found here: TCGA (http://portal.gdc.cancer.gov/) and GEO (www.ncbi.nlm.nih.gov/) under the accession number GSE17538 and GSE41258.

## Abbreviations

CRC: Colorectal cancer
TAM: Tumor-associated macrophage
MRG: Macrophage-associated gene
TME: Tumor microenvironment
DEG: Differentially expressed gene
TCGA: The Cancer Genome Atlas Program
GEO: Gene Expression Omnibus
LASSO: Least absolute shrinkage operator
CSC: Cancer stem cell
TIDE: Tumor Immune Dysfunction and Exclusion
FDR: False discovery rate
SVR: Support vector regression
GO: Gene Ontology
KEGG: Kyoto Encyclopedia of Genes and Genomes
ROC: The receiver operating characteristic
AUC: The area under the curve
PCA: Principal component analysis
OS: Overall survival
PFS: Progression-free survival
K-M: Kaplan-Meier
MAF: Mutation annotation format
GSEA: Gene set enrichment analysis
qRT-PCR: quantitative real-time polymerase chain reaction
