# Supplementary material for: Identification and verification of an eight-gene prognostic signature for colorectal cancer based on tumor-associated macrophages
Source: BMC Cancer. 2026 Apr 6;26:635. doi: 10.1186/s12885-026-15965-9 (PMC13188327; doi:10.1186/s12885-026-15965-9)
Supplement: Supplementary file 1 — Supplementary Material 1. [file 12885_2026_15965_MOESM1_ESM.docx]

Supplementary table 1. Primer sequences for MRPS7 and ORC1

| Gene symbol | Forward primer | Reverse primer |
| --- | --- | --- |
| MRPS7 | 5′- TGGAGGAGCTAACTGAGGAGGAGAA-3 | 5′- CACAGAACTTGTTTTCCCTGCTGGA-3′ |
| ORC1 | 5′- CTCAAGCCTAGAACGCCACGTT-3′ | 5′- GGAAGAGACTCAGGTACAGCAG-3′ |
| GAPDH | 5′- ATCACTGCCACCCAGAAGAC-3′ | 5′- TTTCTAGACGGCAGGTCAGG-3′ |
