# Supplementary material for: Identification and verification of an eight-gene prognostic signature for colorectal cancer based on tumor-associated macrophages
Source: BMC Cancer. 2026 Apr 6;26:635. doi: 10.1186/s12885-026-15965-9 (PMC13188327; doi:10.1186/s12885-026-15965-9)
Supplement: Supplementary file 2 — Supplementary Material 2. [file 12885_2026_15965_MOESM2_ESM.docx]

Supplementary Figure 8d: Original Blot


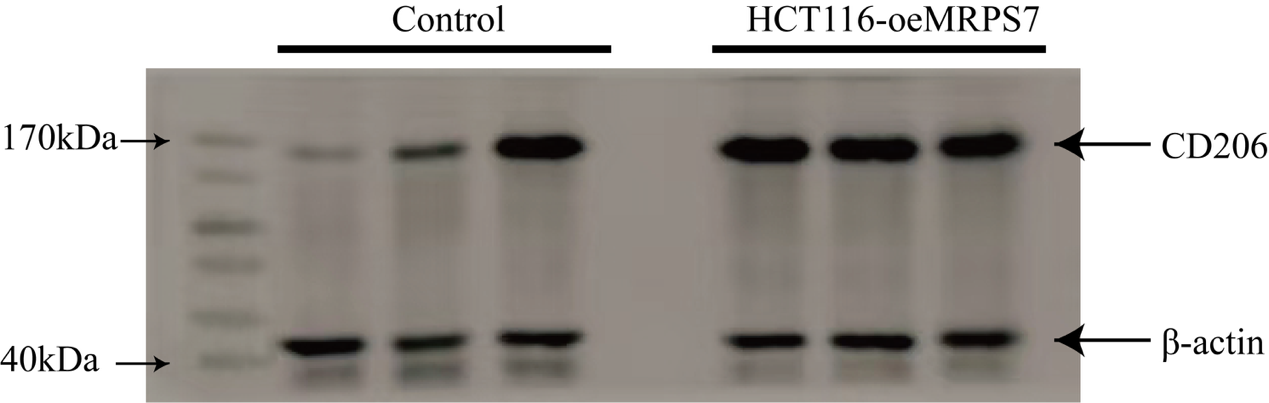


Full Blot 1 (Original image for Figure 8d - Top)

Target Proteins: CD206 (approx. 170 kDa) and β-actin (approx. 42 kDa).

Cell Line: HCT116 colorectal cancer cells.

Experimental Groups: Lanes 1–3: Control (Empty vector). Lanes 4–6: HCT116-oeMRPS7 (MRPS7 overexpression).

Molecular Weight Marker: Indicated on the left (spanning from 40 kDa to 170 kDa).


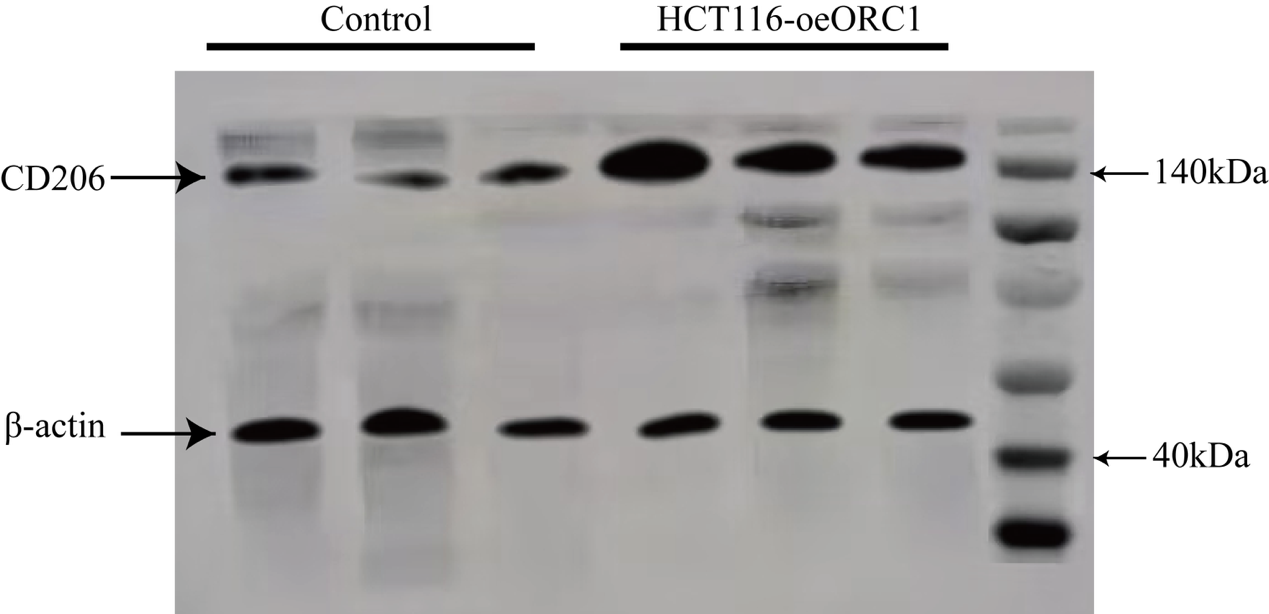


Full Blot 2 (Original image for Figure 8d - Bottom)

Target Proteins: CD206 (approx. 170 kDa/140 kDa marker nearby) and β-actin (approx. 42 kDa).

Cell Line: HCT116 colorectal cancer cells.

Experimental Groups: Lanes 1–3: Control (Empty vector). Lanes 4–6: HCT116-oeORC1 (ORC1 overexpression).

Molecular Weight Marker: Indicated on the left (highlighting 40 kDa and 140 kDa positions).
